# Use of Oxygen-Enriched Oil Dressings in Wound Management: A Scoping Review

**DOI:** 10.3390/jcm15176770

**Published:** 2026-08-31

**Authors:** Sara Sandroni, Valerio Della Bella, Alessandro Sili, Jacopo Fiorini

**Affiliations:** 1 Department of Biomedicine and Prevention, University of Tor Vergata, 00133 Rome, Italy; sarasandroni17@gmail.com; 2 Department of Nursing Professions, University Hospital of Tor Vergata, Viale Oxford, 81, 00133 Rome, Italy; 3 Italian Evidence-Based Practice and Health Research Center, JBI Centre of Excellence, 00136 Rome, Italy

**Keywords:** advanced wound dressing, oxygen-enriched oil, wound healing, scoping review

## Abstract

**Background/Objectives**: Oxygen-enriched oils represent an emerging class of advanced dressings based on the controlled release of reactive oxygen species, with the potential to modulate the wound microenvironment and support reparative processes. Despite their increasing clinical use, the available evidence remains fragmented, and no comprehensive synthesis is currently available on their fields of application and associated outcomes. **Methods**: A scoping review was conducted in accordance with the Joanna Briggs Institute methodology and the PRISMA-ScR reporting guidelines. PubMed, CINAHL, Scopus, and Web of Science were searched without time restrictions. Studies conducted in patients and evaluating oxygen-enriched oil-based dressings for the management of skin wounds were included. Two independent reviewers selected the studies, extracted and narratively synthesized the data, and performed the quality appraisal. **Results:** The search identified 142 records; after the selection process, nine studies including a total of 501 patients were included. The studies comprised different designs, ranging from randomized controlled trials to case reports. Applications included pediatric postoperative wounds (distal hypospadias and pilonidal disease), postsurgical wounds related to hidradenitis suppurativa, angular cheilitis, and oncological wounds. The most frequently investigated outcomes included reductions in healing time, pain, local complications, and infectious burden, as well as improvements in scar quality and patient satisfaction. **Conclusions**: Oxygen-enriched oils represent a promising wound-care strategy in different clinical settings. Although preliminary findings are favorable, independent controlled studies with larger samples and standardized outcomes are needed to define their comparative effectiveness and role in clinical practice more precisely.

## 1. Introduction

Skin wounds represent a major challenge for healthcare systems worldwide, affecting approximately 6.5 million people each year in the United States [1,2] and 1.47 individuals per 1000 population in the United Kingdom [1]. Moreover, having a wound requires individuals to modify their habits and lifestyle and inevitably reduces perceived quality of life (QoL) [3,4]. Beyond their QoL impact, skin wounds also represent a significant burden for healthcare systems in terms of care-related costs, use of healthcare resources, and prolonged care pathways [5]. Wound management requires complex interventions, prolonged follow-up and frequent dressing changes, with a substantial organizational and managerial impact on healthcare services. Wound management remains clinically challenging. In the presence of comorbidities, wounds often require complex dressings [2,6] and might heal over prolonged periods, in some cases beyond four weeks, thereby becoming chronic wounds [7].

Among the different types of wounds, pressure wounds had a prevalence ranging from 3% to 31% in long-term care or primary-care settings [8]. Venous wounds were more common in women [9] and vary according to geographical area and care setting [10]. Global epidemiological studies remained limited and identified a pooled prevalence of venous wounds of approximately 0.32% and a pooled incidence of approximately 0.17% [11]. Lastly, diabetic foot wounds affect approximately 18.6 million people worldwide each year [12].

Despite the etiological heterogeneity of skin wounds, pathophysiological factors might contribute to delayed healing, including persistent hypoxia, chronic inflammation, altered fibroblast activity, redox imbalance, and the presence of biofilm [13,14]. These conditions impair the progression of the reparative process and provide the rationale for developing therapeutic strategies aimed at controlling the wound microenvironment.

In this clinical scenario, tissue oxygenation plays a fundamental role in repair processes, as it supports cellular metabolism, collagen synthesis, angiogenesis, and immune defense mechanisms [15,16]. Several wound-care frameworks, including the TIME model (tissue, infection/inflammation, moisture balance, edge of wound), have been developed to guide wound assessment and management and to promote progression toward the proliferative and remodeling phases of healing [17,18,19].

Several devices have also been implemented to improve tissue oxygenation and support tissue repair in wounds, thereby helping to prevent chronicity [20,21]. Among the strategies proposed to support management of the wound microenvironment, increasing attention has been directed toward oxygen-enriched oils (OEOs) [22,23,24,25]. These devices, available in different formulations and dressing systems, have progressively entered clinical practice based on the hypothesis that controlled release of reactive oxygen species (ROS) might contribute to modulation of the wound microenvironment and support reparative processes [26]. Restoration or maintenance of adequate oxygen levels might modulate the wound microenvironment, promote tissue repair processes [13,26], and counteract persistent hypoxia and chronic inflammation [27].

Despite the growing clinical OEO use, the available evidence remained heterogeneous across wound types, populations, and care settings [28,29]. Published studies included surgical wounds, oncological wounds, and other dermatological conditions, with wide heterogeneity in the devices used, outcomes assessed, and study designs adopted. This fragmentation led to a difficulty in determining which clinical areas have been most extensively studied, which benefits have been reported, and which areas require further investigation [30]. Furthermore, to date, no synthesis of the literature was available that maps the fields of application and evidence relating to OEO use across different types of skin wounds. The objective of this scoping review was to map the available evidence on the use of oxygen-enriched oils in the management of skin wounds, describing the clinical contexts of use, intervention characteristics, reported outcomes, and areas requiring further research.

The review question was as follows: what evidence is available regarding the use of oxygen-enriched oils in the management of skin wounds?

## 2. Materials and Methods

### 2.1. Review Design

A scoping review of the literature was conducted between November 2025 and March 2026, according to the Joanna Briggs Institute (JBI) Manual [31] and Preferred Reporting Items for Systematic Reviews and Meta-Analyses extension for Scoping Reviews [32] (Supplementary File S4). The protocol of this review was registered on the Open Science Framework (https://osf.io/b64se) [33].

### 2.2. Eligibility Criteria and PCC Framework

The research question was developed using the PCC framework (population, concept, context). The population (P) comprised humans with skin wounds/wounds of any nature and etiology (e.g., acute or chronic). Animal and in vitro studies were excluded. Oral mucosal wounds or wounds were not included because they are characterized by healing mechanisms, tissue microenvironments, and therapeutic strategies that differ from skin wounds. This choice was made to maintain greater clinical homogeneity in the included population. The concept (C) focused on OEO use in skin wounds. Oxygenated oils are technologically advanced compounds that tend to release ROS onto the wound, supporting tissue repair. OEO is available in multiple formulations, like gels or in scaffolds made of other materials, provided that the product functions as a primary, non-composite dressing.

The context (C) included any healthcare setting, such as hospitals or community-care facilities. No geographical restrictions were applied.

This scoping review considered and included, in order of methodological priority, randomized controlled trials (RCTs), quasi-randomized controlled studies, retrospective and prospective cohort studies, case-control studies, and analytical cross-sectional studies. In the absence of experimental studies, descriptive cross-sectional studies, case series, and individual case reports were also considered for inclusion. Literature reviews and discursive papers were excluded. To ensure a comprehensive evaluation of the topic, manuscripts written in any language were eligible for inclusion. When manuscripts were written in languages other than the researchers’ native languages (e.g., Chinese), artificial intelligence tools were used to support the translation process.

### 2.3. Search Strategy

The search was conducted in MEDLINE (via PubMed), the Cumulative Index to Nursing and Allied Health Literature (CINAHL), Scopus, and Web of Science (WoS). No time limits were applied. The following keywords were used: oxygen-enriched oil, hyperoxygenated fatty acid, oxygen oil, and enriched oil. The English term wound was incorporated in all possible variants identified from the harmonized glossary of wound-care terms [34]. First, MeSH terms for the identified keywords were searched. Subsequently, a search string was created for each PCC element by combining synonyms and Boolean operators. The search string was entered into the selected databases, and a supplementary file was created to ensure complete retrieval of the available literature (Supplementary File S1).

### 2.4. Selection of Studies

Records retrieved from the databases were imported into Rayyan (https://new.rayyan.ai/) to support the selection process. After duplicate removal, two researchers (SS, JF) selected studies independently, in a blinded and simultaneous manner, following the PCC framework and predefined criteria. Titles were assessed first, followed by abstracts and then full texts. In cases of disagreement, a third researcher (VDB) was involved.

### 2.5. Data Extraction and Analysis

In accordance with the JBI Manual, a data-extraction table (Supplementary File S2) was developed to summarize the sources extracted and included in the review, considering: title, author(s), year and country, aim, study design, sample, results, and conclusions. The extracted data provided an overview of possible treatments for skin wounds with OEO, facilitating a comprehensive understanding of the phenomenon and of potential clinical treatment options across the different types of skin wounds involved. Data were synthesized according to sample type, wound treated, and OEO mode of application.

### 2.6. Quality Appraisal

Two independent reviewers (SS, JF) critically appraised the studies included in the review using the JBI critical appraisal tools according to the design of each included study. In cases of disagreement, a third researcher (VDB) was involved. Studies were rated as high quality when more than 80% of criteria were met, as moderate quality when they scored 50–80%, and low quality when they scored below 50%. In accordance with JBI methodology for scoping reviews, no studies were excluded based on quality appraisal.

## 3. Results

The methodological quality of the manuscripts was assessed using JBI appraisal tools [31] and according to the declared study design. The quality appraisal revealed considerable heterogeneity in methodology and reporting of results. Overall, the methodological quality of the included studies ranged from low to high, varying between 30% and 92.3% across different study designs. RCTs showed the highest methodological quality, with limited critical issues mainly related to blinding of professionals or participants. Observational studies, case reports, and case series showed variable methodological quality, with shortcomings in the description of inclusion criteria, characterization of the clinical setting, management of potential confounding factors, and description of statistical analyses. In addition, potential bias and limited independent validation might arise from the concentration of studies conducted by the same research group (Supplementary File S2).

**Figure 1:**
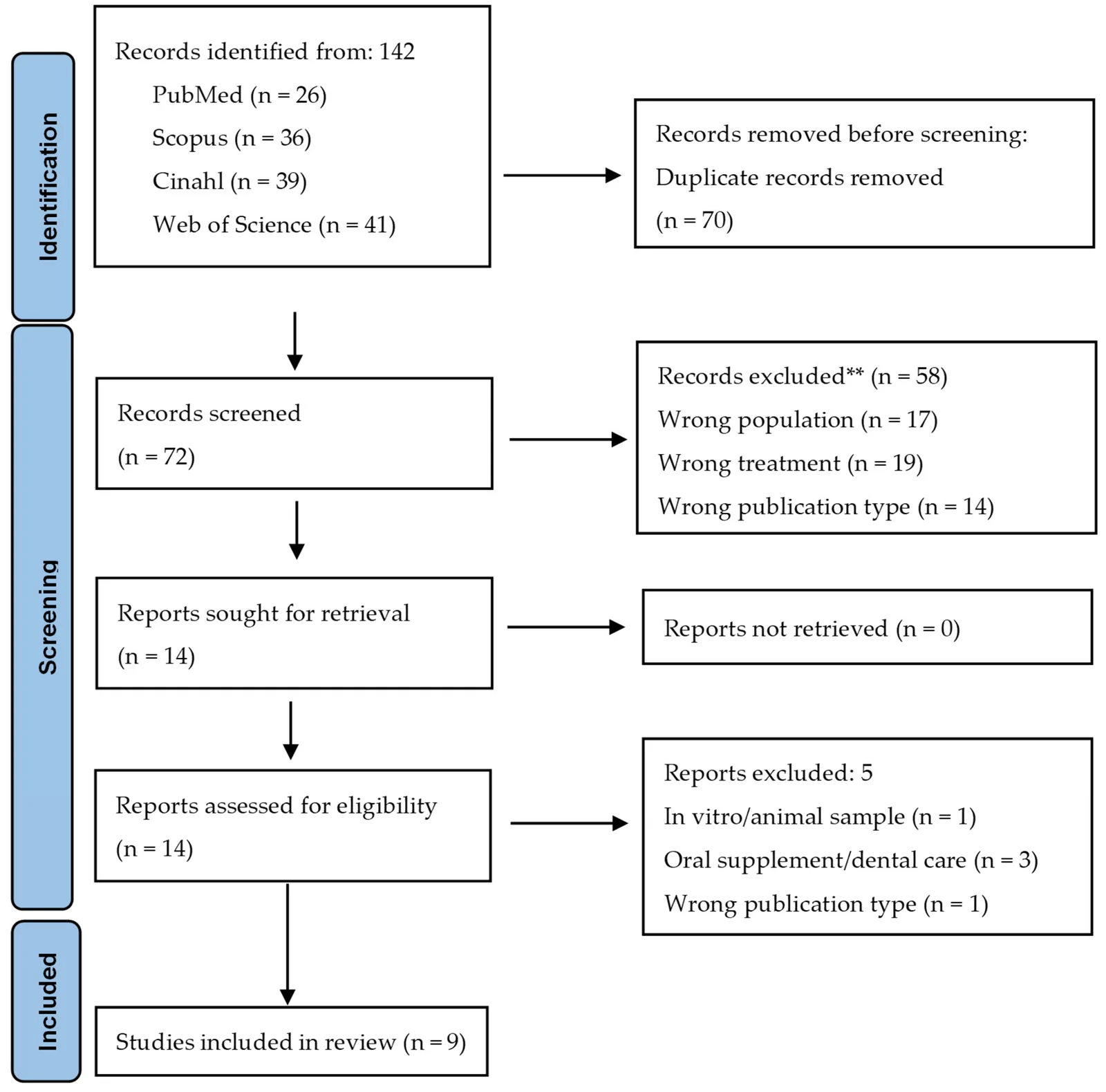
PRISMA flow diagram of the conducted review process.

The search identified 142 records from the databases. After duplicate removal, 72 articles were screened by title and abstract. Of these, 58 studies were excluded because they did not meet the PCC criteria, due to an inappropriate population, inconsistent treatment, ineligible publication type, or animal/in vitro experimentation. Subsequently, 14 full-text reports were assessed for eligibility. Five studies were excluded because they focused on dental/oral applications, were conducted on animals, or had inappropriate publication types (e.g., literature review). Nine studies [28,29,30,35,36,37,38,39,40] were finally included in the synthesis (Figure 1).

The studies were mainly conducted in Italy (Supplementary File S3), including 501 pediatric [28,30,36,37,39] and adults patients [29,35,38,40], and showed marked heterogeneity in study design, wound type, device formulation, and outcomes assessed [28,29,30,35,36,37,38,39,40]. The study designs included a single case report [35], case series [38], RCTs [28], and observational studies. Pediatric studies concerned postoperative wounds of distal hypospadias or pilonidal disease. Adult studies assessed postsurgical wounds, hidradenitis suppurativa, angular cheilitis, and locally advanced breast cancer wounds. OEO formulations were also heterogeneous: gels, matrices, dressing pads, preformed dressings, tubular devices, and impregnated scaffolds. Regarding investigated outcomes, the most frequently assessed outcomes were wound healing, local complications, pain, infection, patient satisfaction, and in some studies, QoL (Table 1).

Two RCTs evaluated OEO use in the postoperative pediatric distal hypospadias [28,30]. In the first study, an OEO gel was compared with a hyaluronic acid cream. Mean healing time was 15.8 days in the OEO group compared with 27.5 days in the control group (*p* = 0.001). The incidence of preputial dehiscence was 0/57 in the experimental group and 5/57 in the control group (*p* = 0.001), whereas no significant differences were observed in the urethrocutaneous fistula rate. In the second study, an internally OEO-coated tubular device was evaluated against a traditional dressing associated with gel. Mean healing time was 14.2 days in the experimental group compared with 18.5 days in the control group (*p* = 0.001), while postoperative complications were reported in 9.3% of patients in the control group and in no patients in the experimental group (*p* = 0.001). Nurses also assigned the tubular device a median management-difficulty score of 1.2 compared with 3.5 in the control group (*p* = 0.001). Three studies addressed the management of pilonidal disease in pediatric patients [36,37,39]. Two retrospective studies evaluated multimodal protocols including OEO use in association with minimally invasive surgical procedures and laser epilation programs. In one study of 127 patients undergoing pediatric endoscopic pilonidal sinus treatment, mean healing time was 21 days compared with 29 days in the silver sulfadiazine group (*p* = 0.001); recurrence was observed in 1/72 patients compared with 5/55 patients (*p* = 0.001). In a second study of 87 patients, mean healing time was 21 days compared with 28.1 days (*p* = 0.001), while recurrence was observed in 1/47 patients compared with 6/40 patients (2.1% vs. 15%; *p* = 0.001). In the same study, wound infection was reported in 1/47 patients in the experimental group and 4/40 in the control group (2.1% vs. 10%; *p* = 0.001). A further descriptive study reported OEO use in three pediatric patients undergoing open surgery, describing wound healing within five weeks, absence of recurrence at six months, and only one minor complication represented by mild local hyperemia.

Michelucci et al. [29] evaluated three different strategies for the management of postsurgical wounds related to hidradenitis suppurativa, including an OEO dressing. The outcomes considered included wound area, wound bed score (WBS), and perceived pain during follow-up. In the OEO group, mean wound area decreased from 18.51 cm^2^ to 3.38 cm^2^, and mean pain score decreased from 6.0 to 0.9 after four weeks. Similar improvements were observed in the control group, with no statistically significant differences between the strategies compared. A further case report [35] described the OEO dressing in an older female patient with a postoperative wound of the lower limb. The wound had an initial area of 16.2 cm^2^ with local signs of infection; clinical follow-up over 103 days documented progressive reduction in wound surface area, with progressive resolution of the inflammatory and infectious signs described by the authors.

Arduino et al. [38] evaluated OEO gel for the treatment of angular cheilitis in 30 adults. After ten days of treatment, median wound size decreased from 5.0 mm to 0.0 mm (*p* < 0.001), while median pain score decreased from 3.0 to 0.0 (*p* < 0.001). Microorganism colonization decreased from 30/30 patients at baseline to 5/30 at the end of treatment. The study also reported good treatment tolerability and absence of relevant adverse events.

Casella et al. [40] evaluated an OEO matrix integrated into a scaffold dedicated to the local management of locally advanced breast cancer wounds. After 30 days of treatment, the wound area decreased from 178.6 cm^2^ to 62.3 cm^2^. Mean pain decreased from 7.8 to 2.6 in the group treated with the experimental device and from 7.9 to 5.2 in the control group. Improvements in exudate, odor, and wound-management-related QoL were also reported.

## 4. Discussion

This scoping review was conducted to map the available evidence on OEO use in the management of skin wounds. Despite the heterogeneity of the evidence, the included studies reported reductions in wound area [29,38], pain [28,38] and complications [40], accelerated healing [36], colonization control [35,39] and good tolerability [36]. The concept of reactive species modulation involved the ROS-controlled release, differing substantially from other devices that incorporate large quantities of ozone, which, being highly unstable, tends to decompose immediately into reactive compounds and exhaust its action rapidly.

The included studies reported applications across different settings, including pediatric surgery and dermatology, involving both adult and pediatric populations. Wound types were also variable and included postsurgical wounds and neoplastic wounds. The review findings could be interpreted in accordance with the TIME framework, which is widely referenced in the literature on wound-bed preparation. OEO’s action might be related to several dimensions of this model: colonization control, moisture regulation and support for epithelial edge progression. In the study by Michelucci et al., the device is explicitly discussed as a possible component that could be integrated into the TIME model for postsurgical wounds [29]. The case report by Guarro et al. might also be considered from this perspective. In an older female patient with a postoperative wound of the lower limb, initially inflamed, infected, and measuring 16.2 cm^2^, OEO pad use was associated with wound area reduction, disappearance of periwound inflammation, and reduced colonization at follow-up [35]. As this was a single case, the finding retains descriptive value, while remaining consistent with the hypothesis that the device might support local control of factors that hinder repair.

Distal hypospadias represented the setting with the most methodologically robust evidence. The two RCTs reported concordant findings in favor of OEO treatment, both as a gel and as the internal coating of a tubular device [28,30]. The observed outcomes concern healing speed, edema, some local complications, scar quality, ease of management, and costs. The interpretation appears biologically and clinically plausible in an anatomical site exposed to moisture, contamination, and mechanical traction. However, transferability of these data requires caution. The studies involved selected pediatric patients with distal hypospadias and specific surgical protocols; therefore, the results could not be automatically extended to proximal forms or different surgical contexts. In addition, the trial on the tubular device had a follow-up limited to 60 days, a factor that the authors themselves recognized as potentially insufficient to detect some late complications of urethroplasty [28].

The available studies indicated that OEO might represent an element of interest within structured protocols. In the studies by Esposito et al. on the surgical management of pilonidal cysts, lower recurrence, shorter healing time, and greater satisfaction were reported; however, the retrospective design and concurrent use of laser epilation required caution when attributing outcomes solely to the device [36,37]. The case series by Bisol et al., involving three pediatric patients undergoing open surgery for chronic pilonidal disease, described a favorable course, with completion of the healing process and return to activities within a mean follow-up of five weeks, absence of recurrence at six months, and only one mild event of local skin hyperaemia [39]. This contribution was useful from a descriptive and hypothesis-generating perspective but did not allow comparative inferences.

The study by Michelucci et al. extends observation to postsurgical wounds related to hidradenitis suppurativa (HS). After four weeks, all three treatments compared showed favorable within-group changes in wound area, WBS and pain; in the OEO group, clinically relevant reductions in area and pain were recorded but without statistically significant differences compared with the other strategies [29]. At present, the device might therefore be considered a possible option to be integrated into advanced wound management, without demonstrated superiority over comparator treatments.

In this setting, Arduino et al. evaluated OEO use in 30 patients with angular cheilitis. After 10 days of application, the authors reported a significant reduction in perceived pain, maximum wound size, and microbial burden, with no adverse reactions or tolerability problems [38]. The methodological quality of the series was higher than that of the other descriptive studies included; however, the absence of randomization and of a therapeutic comparator did not allow the specific effect of the intervention to be quantified with certainty.

The study by Casella et al. concerned a particularly complex clinical setting in which local treatment often had a palliative or supportive purpose rather than a truly curative one. In patients treated with a cup device and OEO, the authors described, after 30 days, reductions in area, exudate, and odor, together with improved QoL compared with the control group [40]. However, the authors themselves noted that the small sample size and observational nature of the study did not permit definitive conclusions. Use in neoplastic wounds should therefore be considered preliminary.

### 4.1. Limitations

The results of this review must be interpreted considering some limitations. The included literature described a field of research still characterized by a limited number of studies, marked heterogeneity, and non-uniform methodological quality.

A first source of bias was the small sample size of a relevant proportion of the studies, including a single case report [35], three-case series [39], and a twenty-participant observational study [40]. Limited samples did not allow reliable effect estimates or adequate assessment of adverse events. Even in studies with larger samples, limitations of precision and transferability remained. In particular, they enrolled selected samples, involved specific surgical procedures, and tested the efficacy of the treatment in a multimodal protocol.

A second relevant limitation concerned the presence of confounders that were not fully controlled. This was particularly evident in studies on pediatric pilonidal disease, in which OEO use was integrated with other procedures [36,37]. The observed reduction in recurrence and shortening of healing time in these studies might reflect the combined effect of the entire therapeutic pathway, making it difficult to attribute the outcome to the OEO device alone.

A third potential source of bias was the concentration of a substantial proportion of the scientific output within a small number of research groups. Several included studies came from recurring author groups and were conducted in the same country [28,30,36,37], thereby limiting the independence of the available confirmations and increasing the risk that results reflect organizational, technical, or interpretative specificities that have not yet been validated in external contexts.

Further limitations were attributable to clinical and methodological heterogeneity. Studies differed in pathophysiology, anatomical site, microbial contamination, tissue regenerative potential, therapeutic objectives, and spontaneous probability of healing. Heterogeneity also concerned the interventions. The devices examined were not interchangeable: gels, pads, and preformed structures such as a tubular device were used and applied heterogeneously in terms of dressing-change frequency, treatment duration, and association with other products. This clinical heterogeneity complicated direct comparison between studies and limits the possibility of constructing a synthesis of effect.

Finally, this scoping review had limitations inherent to its design. First, no quantitative synthesis of effects was planned. This choice was consistent with the exploratory nature of the review and with the clinical and methodological heterogeneity of the included studies, but it prevented the effectiveness synthesis and weighted comparisons between interventions.

### 4.2. Implications for Clinical Practice

The most strongly supported clinical context was pediatric distal hypospadias surgery, where favorable outcomes were associated with OEO use [28,30]. However, the specific population and surgical setting limited the generalizability of these findings.

In pediatric pilonidal disease, the available studies suggested a possible benefit of OEO matrices for the quality of the postoperative course [36,37]. However, treatment was embedded within multimodal protocols such as laser epilation, making it difficult to establish with certainty the specific effect of the device.

In adults, implications for clinical practice should be even more cautious. The study on postsurgical wounds related to hidradenitis suppurativa (HS) documented favorable within-group changes but did not demonstrate OEO superiority [29]. Similarly, the prospective study on angular cheilitis showed positive outcomes, but the absence of a control group and the exploratory nature of the design limited the findings [38].

### 4.3. Directions for Future Research

The main gap in the literature concerned the limited availability of independent, multicenter, comparative studies. Moreover, a substantial proportion of the evidence came from small samples. Reproducibility of the findings in different care contexts remained to be demonstrated. Evidence for chronic wounds remained limited and there was also a lack of outcome standardization and of direct comparisons with other advanced dressings commonly used in clinical practice.

Future studies should adopt shared core outcomes such as time to complete healing or percentage reduction in wound area. This would facilitate comparison between studies and, in the longer term, enable reliable meta-analyses.

Further research should clarify the relationship between device formulation, ROS-release modality, dressing-change frequency, and clinical response. It would be useful to distinguish effects attributable to redox modulation from those related to the protective or mechanical function of the support, especially in studies using complex devices or associated interventions.

## 5. Conclusions

This scoping review reconstructed the state of evidence on the use of oxygen-enriched oils in wound management. The included studies described applications across different clinical contexts, with a predominance of experiences in pediatric surgery and fewer contributions related to complex adult wounds, mucocutaneous wounds, and wounds in oncology settings.

Overall, preliminary and favorable findings were found regarding local healing, control of some postoperative outcomes, and for specific populations. However, considering the different levels of evidence, independent, comparative, and methodologically robust studies are needed to evaluate standardized outcomes and long-term effects and to explore different clinical contexts.

## Figures and Tables

**Table 1:** Synthesis of the included articles.

Authors	Sample	Dressing Composition	Wound Aetiology and Site	Evaluated Outcomes							
				Area	Pain	Comfort	HT	Inf	Recur	Compl	Satisf
Guarro et al., 2023 [35]	1 adult	Hyperoxidized oil-based medical device pad	Postoperative wound of the lower limb	+	nr	nr	+	nr	nr	nr	nr
Michelucci et al., 2023 [29]	25 adults	Oxygen-enriched olive oil in the form of an oily gel in a prefilled syringe	Postsurgical wounds related to hidradenitis suppurativa located in the groin, axilla, pubic region, and gluteal region	+	+	+/−	nr	nr	nr	nr	+/−
Bisol et al., 2022 [39]	3 pediatric patients	OEO and hyaluronic acid gauze	Surgical wound in pilonidal disease located in the gluteal cleft and sacrococcygeal region	−	−	−	−	+	+	+	+
Esposito, Del Conte, Esposito et al., 2021 [37]	87 pediatric patients	OEO-based gel with wet gauze composed of a hyaluronic acid dressing	Surgical wound in pilonidal disease located in the gluteal cleft and sacrococcygeal region	−	+	−	+	+	+	+	−
Arduino et al., 2021 [38]	60 adult patients	OEO-based gel	Angular cheilitis at the corners of the lips	+	+	+	+	+	−	−	−
Esposito, Coppola, Del Conte et al., 2021 [28]	64 pediatric patients	OEO-based gel and wet gauze and a tubular device with oxygen-enriched olive oil	Surgical wound after distal hypospadias repair	+	nr	−	+	+	+	+	+
Esposito, Del Conte, Cerulo et al. 2021 [30]	114 pediatric patients	OEO-based gel	Surgical wound after distal hypospadias repair	+	nr	+	+	+	+	+	−
Esposito et al., 2020 [36]	127 pediatric patients	OEO-based gel dressing	Surgical wound in pilonidal disease	+	+	+	+	+	+	+	+
Casella et al., 2021 [40]	20 adult patients	OEO matrix gel and a preformed cup device in polyester with polyurethane soaked in an oxygen-enriched oleic matrix	Skin wounds associated with locally advanced breast cancer	+	+	+	nr	+	nr	nr	nr

Note: +: statistically significant impact and improvement in the outcome; −: non-statistically significant impact and no improvement in the outcome; +/−: uncertain result due to borderline statistical significance and/or slight improvement in the outcome; nr: outcome not reported or not included in the study. OEO = oxygen-enriched oil; HT = healing time; Inf = infection; Satisf = satisfaction; Recur = recurrence; Compl = complications.

## Data Availability

No new data were created or analyzed in this study. Data sharing is not applicable to this article.

## Supplementary Materials

The following supporting information can be downloaded at: https://www.mdpi.com/article/10.3390/jcm15176770/s1. Supplementary File S1: search strategy; Supplementary File S2: quality appraisal oil; Supplementary File S3: extraction table of included articles; Supplementary File S4: PRISMA Checklist.

## Abbreviations

The following abbreviations are used in this manuscript:

CINAHL	Cumulative Index to Nursing and Allied Health Literature
JBI	Joanna Briggs Institute
OEO	Oxygen-enriched oil
QoL	Quality of life
HS	Hidradenitis suppurativa
PCC	Population, concept, context
RCT	Randomized controlled trial
ROS	Reactive oxygen species
TIME	Tissue, infection/inflammation, moisture balance, and edge of wound
WoS	Web of Science
