# Supplementary material for: Use of Oxygen-Enriched Oil Dressings in Wound Management: A Scoping Review
Source: J Clin Med. 2026 Aug 31;15(17):6770. doi: 10.3390/jcm15176770 (PMC13566079; doi:10.3390/jcm15176770)
Supplement: Supplementary file 1 [file jcm-15-06770-s001.zip › Supplementary file S1_search strategy.pdf]

Supplementary file 1: Search strategy

Search conducted on the 15 December 2025

**Database**	**Query**	**Records**
PubMed	("Oxygen enriched oil*" OR "Hyper oxygenated fatty acid*" OR "oxygen oil*" OR "enriched oil*") AND ("Wounds and Injuries"[Mesh] OR wound* OR injur* OR "Ulcer"[Mesh] OR "ulcer*" OR "lesion*" OR "Dehiscence*")	N= 26
Scopus	(TITLE-ABS-KEY ("Wounds and Injuries" OR wound* OR injur* OR "Ul[illegible] OR "ulcer*" OR "lesion*" OR "Dehiscence*")) AND (TITLE-ABS-KE[illegible] "Oxygen enriched oil*" OR "Hyper oxygenated fatty acid*" OR "oxygen oil*" [illegible] "enriched oil*"))	N = 36
WOS	("Oxygen enriched oil*" OR "Hyper oxygenated fatty acid*" OR "oxygen oil*" OR "enriched oil*") AND ("Wounds and Injuries" OR wound* OR injur* OR "Ulcer" OR "ulcer*" OR "lesion*" OR "Dehiscence*")	N = 41
CINAHL	(("Oxygen enriched oil*" OR "Hyper oxygenated fatty acid*" OR "oxygen oil*" OR "enriched oil*")) AND (("Wounds and Injuries" OR wound* OR injur* OR "Ulcer" OR "ulcer*" OR "lesion*" OR "Dehiscence*"))	N = 39
