# Supplementary material for: Use of Oxygen-Enriched Oil Dressings in Wound Management: A Scoping Review
Source: J Clin Med. 2026 Aug 31;15(17):6770. doi: 10.3390/jcm15176770 (PMC13566079; doi:10.3390/jcm15176770)
Supplement: Supplementary file 1 [file jcm-15-06770-s001.zip › Supplementary file S2_ quality appraisal oil.pdf]

Supplementary file. Quality appraisal results.

JBI CRITICAL APPRAISAL TOOL FOR assessment of risk of bias for randomized controlled trials 2023

Barker TH, Stone JC, Sears K, Klugar M, Tufanaru C, Leonardi-Bee J, Aromataris E, Munn Z. The revised JBI critical appraisal tool for the assessment of risk of bias for randomized controlled trials. JBI Evidence Synthesis. 2023;21(3):494-506

Authors	Year	1) Was true randomization used for assignment of participants to treatment groups?	2) Was allocation to treatment groups concealed?	3) Were treatment groups similar at the baseline?	4) Were participants blind to treatment assignment?	5) Were those delivering the treatment blind to treatment assignment?	6) Were treatment groups treated identically other than the intervention of interest?	7) Were outcome assessors blind to treatment assignment?	8) Were outcomes measured in the same way for treatment groups?	9) Were outcomes measured in a reliable way	10) Was follow up complete and if not, were differences between groups in terms of their follow up adequately described and analysed?	11) Were participants analysed in the groups to which they were randomized?	12) Was appropriate statistical analysis used?	13) Was the trial design appropriate and any deviations from the standard RCT design (individual randomization, parallel groups) accounted for in the conduct and analysis of the trial?	Quality appraisal Score
Esposito C., Del Conte F., Cerulo M. et al.	2021	y	y	y	y	u	y	y	y	y	y	y	y	y	92.3%
Esposito C., Coppola V., Del Conte F. et al.	2021	y	y	y	u	n	y	y	y	y	y	y	y	y	84.6%

Note: y = yes; n = no; u = unclear.

JBI Critical Appraisal tools for use in JBI Systematic Reviews Checklist for analytical cross sectional studies

Moola S, Munn Z, Tufanaru C, Aromataris E, Sears K, Sfetcu R, Currie M, Qureshi R, Mattis P, Lisy K, Mu P-F. Chapter 7: Systematic reviews of etiology and risk . In: Aromataris E, Munn Z (Editors). JBI Manual for Evidence Synthesis. JBI, 2020.

Authors	Year	1) Were the criteria for inclusion in the sample clearly defined?	2) Were the study subjects and the setting described in detail?	3) Was the exposure measured in a valid and reliable way?	4) Were objective, standard criteria used for measurement of the condition?	5) Were confounding factors identified?	6) Were strategies to deal with confounding factors stated?	7) Were the outcomes measured in a valid and reliable way?	8) Was appropriate statistical analysis used?	Quality appraisal Score	Comments
Casella et al.	2022	y	u	u	y	n	n	y	n	37.5%	Missing the description of the settings and a section of statistical analysis in the methodology.
Esposito C. et al.	2020	n	u	y	y	n	n	y	y	50%	Inclusion criteria not defined. Missing the description of the settings.
Michelucci A. et al.	2023	n	n	y	y	n	n	y	y	50%	Study design unclear for the presence of randomization and allocation. Inclusion criteria not defined. Missing the description of the settings.
Esposito C., Del Conte F., Esposito G. et al.,	2021	n	n	y	y	n	n	y	y	50%	Inclusion criteria not defined. Missing the description of the settings.

Note: y = yes; n = no; u = unclear.

JBI Critical Appraisal tools for use in JBI Systematic Reviews Checklist for Case Reports

Moola S, Munn Z, Tufanaru C, Aromataris E, Sears K, Sfetcu R, Currie M, Qureshi R, Mattis P, Lisy K, Mu P-F. Chapter 7: Systematic reviews of etiology and risk. In: Aromataris E, Munn Z (Editors). JBI Manual for Evidence Synthesis. JBI, 2020. Available from https://synthesismanual.jbi.global

Authors	Year	1) Were patient's demographic characteristics clearly described?	2) Was the patient's history clearly described and presented as a timeline?	3) Was the current clinical condition of the patient on presentation clearly described?	4) Were diagnostic tests or assessment methods and the results clearly described?	5) Was the intervention(s) or treatment procedure(s) clearly described?	6) Was the post-intervention clinical condition clearly described?	7) Were adverse events (harms) or unanticipated events identified and described?	8) Does the case report provide takeaway lessons?	Quality appraisal Score	Comments
Guarro G. et al.	2023	y	u	y	y	u	y	n	y	62.5%	-

Note: y = yes; n = no; u = unclear

JBI Critical Appraisal tools for use in JBI Systematic Reviews Checklist for Case Series

Munn Z, Barker T, Moola S, Tufanaru C, Stern C, McArthur A, Stephenson M, Aromataris E. Methodological quality of case series studies, JBI Evidence Synthesis, doi: 10.11124/JBISRIR-D-19-00099

Authors	Year	1) Were there clear criteria for inclusion in the case series?	2) Was the condition measured in a standard, reliable way for all participants included in the case series?	3) Were valid methods used for identification of the condition for all participants included in the case series?	4) Did the case series have consecutive inclusion of participants?	5) Did the case series have complete inclusion of participants?	6) Was there clear reporting of the demographics of the participants in the study?	7) Was there clear reporting of clinical information of the participants?	8) Were the outcomes or follow up results of cases clearly reported?	9) Was there clear reporting of the presenting site(s)/clinic(s) demographic information?	10) Was statistical analysis appropriate?	Quality appraisal Score	Comments
Arduino P.G. et al.,	2021	y	y	y	u	u	y	y	y	y	y	80%	-
Bisol M. et al.	2022	n	n	n	u	u	y	y	u	y	n	30%	-

Note: y = yes; n = no; u = unclear
