# Supplementary material for: Use of Oxygen-Enriched Oil Dressings in Wound Management: A Scoping Review
Source: J Clin Med. 2026 Aug 31;15(17):6770. doi: 10.3390/jcm15176770 (PMC13566079; doi:10.3390/jcm15176770)
Supplement: Supplementary file 1 [file jcm-15-06770-s001.zip › Supplementary file S3_ extraction table of included articles.pdf]

TITLE	AUTHORS /YEAR OF PUBLICATION	AIM	STUDY DESIGN	SAMPLE /SETTING	DRESSING CHARACTERISTICS	METHODS	RESULTS	CONCLUSION
Use of hyperoxidized oils-based medical devices for the wound healing in post-surgical patients	Guarro G., Sara Carella S., Cordellini M.<br><br>2023	To evaluate hyperoxidized oils-based medical device pad's effectiveness in a post-surgical patient presenting with persistent injury of the lower limb.	Case report	1 adult patient/Postoperative, outpatient	Hyperoxidized oils-based medical device pad	88-year-old female patient who had previously undergone orthopedic surgery. The woman had an ulcer on her left leg, on the external malleolus.	The progressive clinical examination, helped by a computer software and the TIME-H prognosis evaluation showed a global reduction of the wound area, the total reduction of the perilesional inflammatory area and the complete absence of signs of infection in about 100 days.	Reactive oxygen species (ROS), a crucial element of the normal wound-healing response. The use of ROS-based dressings has been shown to be essential to facilitate the treatment of postoperative wound healing.
Advanced wound management approaches in Hidradenitis Suppurativa postsurgical lesions	Michelucci A., Janowska A., Granieri G., Manzo Margiotta F., Morganti M.,Romanelli M., Dini V.<br><br>2023	To evaluate the postsurgical results obtained on HS wounds treated with a slow-releasing reactive oxygen species (ROS) oleic matrix and to compare them with the results obtained with the medications suggested by HS-TIME and the use of an ultraportable negative pressure wound therapy (NPWT).	Clinical prospective study	25 adult patients/Postoperative, outpatient	Oxygen-enriched olive oil in the form of an oily gel in a prefilled syringe	After surgical procedure the patients were single-blind randomized and divided into three groups. Group 1 consisted of 10 patients treated twice a week with silver hydrofiber and polyurethane foam as a secondary dressing, according to the principles of HS-TIME. Group 2 included 10 patients treated twice a week with oxygen-enriched olive oil in the form of an oily gel in a prefilled syringe. Group 3 consisted of 5 patients treated with ultraportable NPWT changed every week.	After 2 weeks (V1), a reduction in mean area was detected (Group 1: 51.17%; Group 2: 54.51%; Group 3: 55.07%), as well as an improvement in mean WBS (Group 1: 15.25%, Group 2: 16.94%; Group 3: 17.24%) and a decrease in mean pain NRS (Group 1: 54.24%, Group 2: 55%; Group 3: 50%). After 4 weeks (V2) a significant reduction in terms of wound area, an improvement of WBS, and a decrease in pain NRS were detected in all groups (p-value<0.001). In particular, the reduction in mean area was of 79.91% for Group 1, 81.74% for Group 2, and 74.75% for Group 3. The improvement in mean WBS was of 22.88%, 19.49%, and 25.86%, respectively. Mean pain NRS showed a decrease of 71.19% (Group 1), 75% (Group 2), and 73.33% (Group 3). No statistically significant differences were found in terms of reduction in mean area, mean WBS, and mean pain NRS between the three groups analyzed neither at V1 nor at V2.	The absence of statistically significant differences at V1 and V2 among the three groups demonstrates equal effectiveness of the three treatments in terms of wound size reduction, WBS improvement, and pain decrease.
Oxygen-enriched oleic matrix (NovoX) for wound healing in pediatric patients undergoing open surgical treatment for pilonidal disease:	Bisol M., Tykhomyrova S., Pagliara C., Scarpa M., Guida E., Olenik D., Codrich D., Schleef J. and Boscarelli A.	To evaluate the preliminary experience with oxygen-enriched oleic matrix	Case series	3 pediatric patients/ Postoperative, outpatient	Oxygen-enriched oil and hyaluronic acid gauze	All patients enrolled were medicated with oxygen-enriched oil directly applied to the wound and covered with a hyaluronic acid gauze.	All patients (3) underwent definitive surgical excision of a pilonidal cyst using the open surgical technique and dressing with oxygen enriched oleic matrix. Six months after the surgery, no recurrence occurred, and all patients reported high satisfaction regarding the cosmetic result.	The oxygen-enriched oil- based product is easy to apply, safe, and effective for treating pediatric patients undergoing surgical treatment for chronic PD. In particular, it appears to confer slightly faster wound healing with good aesthetic outcomes in patients treated via open surgery followed by healing by secondary intention.

Preliminary experience	2022							
Standardization of Pre- and Postoperative Management Using Laser Epilation and Oxygen-Enriched Oil-Based Gel Dressing in Pediatric Patients Undergoing Pediatric Endoscopic Pilonidal Sinus Treatment (PEPSiT)	Esposito C., Del Conte F., Esposito G.,Coppola V., Cerulo M., and Escolino M.<br><br>2021	To evaluate our current wound treatment protocol (Laser Epilation and oxygen-enriched oil gel dressing) with our previous protocol (silver sulfadiazine spray) and demonstrate its effectiveness as a means to prevent PSD recurrence in children undergoing PEPSiT.	Retrospective analysis	87 pediatric patients/Preoperative, Postoperative, outpatient	Oxygen-enriched oil-based gel wet gauze composed by hyaluronic acid dressing	They retrospectively reviewed the data of 87 pediatric patients, who underwent PEPSiT over a 24-month period. The patients were divided into two groups: G1 (n=47) treated with pre- and postoperative Laser Epilation (LE) and oxygen-enriched oil-based gel dressing; and G2 (n=40) treated only with postoperative dressing using silver sulfadiazine spray.	The median healing time significantly decreased in G1 (21 days) compared with G2 (28.1 days) ($p = 0.001$). The disease recurrence rate was significantly lower in G1 (n=1, 2.1%) compared with G2 (n = 6, 15%) ($p = 0.001$), and the wound infection (abscess) rate was significantly lower in G1 (n = 1, 2.1%) compared with G2 (n=4, 10%) ($p=0.001$).	Patients treated with LE and oxygen-enriched oil-based gel dressing reported faster healing time and lower recurrence and wound infection rates compared with those not receiving the same treatment.
A single-center prospective pilot open-label study to assess the efficacy of topical application of an oxygen-enriched oil for the treatment of angular cheilitis	Arduino P.G., Cabras M., Macciotta A., Longhi F., Gambino A., and Broccoletti R.<br><br>2021	To evaluate the acceptability and clinical efficacy of an oxygen-enriched oil-based gel for treatment of angular cheilitis.	Prospective case series	60 adult patients/Oral medicine unit, outpatient	Oxygen-enriched oil-based	Patients are divided into two groups: (30) treated with an oxygen-enriched oil-based medical, and the control group (30) without this type of dressing. The preparation was applied 3 times daily for 10 days.	Regarding the initial identification of possible involved pathogens, the results of microbiological evaluation differed significantly between the groups. In the group without clinical signs of AC, only four subjects (13.3%) were positive for *Staphylococcus aureus*. In contrast, many different species were identified in the patients with clinical signs of AC: *Staphylococcus aureus* in 8 cases (26.7%), *Candida albicans* in 14 (46.7%), *Escherichia coli* and *Enterobacter aerogenes* in 1 (3.3%). More- over, in some cases, more than 1 species was present: 4 cases (13.3%) with *Staphylococcus aureus* and *Candida albicans*, and 2 (6.7%) with *Candida albicans* and *Escherichia coli*.<br>A significant reduction in the total count of pathogens at the site of infection was observed ($p < 0.001$). The microbiological evaluation were negative for 83.3% after treatment. Above all, there was a significant reduction in the reported outcome of patients, in terms of the VAS score($p < 0.001$). There was also a reduction in lesion dimension after application of the clinical protocol($p < 0.001$). No patient complained about undesirable side effects.	Data showed that this medical preparation facilitated prompt recovery from reported pain, without adverse reactions.

Evaluation of a New Tubular Finger Oxygen-Enriched Oil Inside-Coated Dressing Device in Pediatric Patients Undergoing Distal Hypospadias Repair: A Prospective Randomized Clinical Trial Part II	Esposito C., Coppola V., Del Conte F., Cerulo M., Esposito G., Crocetto F., Castagnetti M., Calignano A., and Escolino M.<br><br>2021	To evaluate the use of a new tubular finger oxygen-enriched oil inside-coated dressing device and its effect on the post-operative outcome of children undergoing distal hypospadias repair.	Randomized clinical trial	64 pediatric patients/postoperative, outpatient	Oxygen-enriched oil-based gel and wet gauze composed by hyaluronic acid, in the first group (G2) and a tubular device where the inner part was coated with oxygen-enriched olive oil and lavender essential oil in the second group (G1)	The patients were randomized in two groups, each of 32 patients, according to the type of post-operative dressing: the treatment group (G1), the control group (G2). After completing the surgical correction of the hypospadias, the area was washed with saline and dried with gauze. In G1 patients, a new medical device in the form of a tubular finger with a closed tip was adopted. In G2 patients, a layer of oxygen-enriched oil-based gel was directly applied on a wet gauze composed by hyaluronic acid, was wrapped around the penis and subsequently covered by an elastic net bandage to obtain hemorrhage compressive effect.	At clinical examination, the wound healing time was significantly shorter in G1 compared with G2 [14.2 vs. 18.5 days] (*p* = 0.001). Wound healing evaluation using SWAS reported a significantly higher rate of normal healing (≤1) in G1 compared with G2 at 7 days (84.4 vs. 59.4%), 14 days (100 vs. 68.8%), 21 days (100 vs. 84.4%) and 30 days (100 vs. 90.6%) follow-up (*p* = 0.001). No significant difference in SWAS scores was found between G1 and G2 (*p* = 0.33) at 60 days follow-up. No adverse skin reactions or allergies to the product occurred in both groups. The evaluation of foreskin retractability showed no significant difference about incidence of post-operative phimosis between the two groups (*p* = 0.87). The post-operative complications rate was significantly lower in G1 (0%) compared with G2 [(3/32 Clavien IIIb) 9.3%] (*p* = 0.001). In two G2 patients (6.2%), foreskin dehiscence occurred whereas, in one G2 patient (3.1%) breakdown of urethroplasty and preputioplasty occurred due to scratching injuries caused by the child on the 5th POD. The dressing management was subjectively evaluated by nurses to be easier in G1 patients (median score 1.2) compared with G2 ones (median score 3.5) (*p* = 0.001). The median treatment costs were significantly lower in G1 (55 eur) compared with G2 (87 eur) (*p* = 0.001).	This new tubular device was very effective in our series in promoting a faster healing of penis wound compared with the standard dressing method.
Evaluation of efficacy of oxygen-enriched oil-based gel dressing in patients who underwent surgical repair of distal hypospadias: a prospective randomised clinical trial	Esposito C., Del Conte F., Cerulo M., Coppola V., Esposito G., Ricciardi E., Crocetto F., Castagnetti M., Calignano A., Escolino M.<br><br>2021	To evaluate the efficacy of oxygen-enriched oil-based gel dressing on wound healing and postoperative outcome in children who underwent distal hypospadias repair.	Prospective randomised clinical trial	114 pediatric patients/postoperative, outpatient	Wet gauze, impregnated with oxygen-enriched oil-based gel in the first group (G1) or hyaluronic acid cream (G2), is wrapped around the penis and covered by an elastic net bandage	The patients were randomized in two groups each of 57 patients, according to the type of medication: oxygen-enriched oil-based gel (G1) and hyaluronic acid cream (G2). After discharge, parents changed the dressing twice a day for 2–3 weeks postoperatively. The patients were evaluated at 7, 14, 21, 30, 60 and 180 postoperative days and thereafter annually.	At the clinical evaluation, the wound healing time was significantly shorter in G1 compared with G2 [15.8 vs 27.5 days] (*p* = 0.001). Wound healing evaluation using SWAS reported a significantly higher rate of normal healing (≤ 1) in G1 com- pared with G2 at 7 days (78.9% vs 38.6%), 14 days (96.5% vs 57.9%), 21 days (100% vs 80.7%) and 30 days (100% vs 87.7%) follow-up (*p* = 0.001). No adverse skin reactions to the product occurred in both groups.<br><br>The 57.9% of G2 patients resulted significantly “troubled” with the use of the dressing whereas all G1 patients were “not troubled” and the wound was easily managed by parents at home.<br><br>Regarding postoperative complications, no significant difference was found in regard to urethrocutaneous fistula rate between G1 [1/57 (1.8%)] and G2 [3/57 (5.2%)] (*p* = 0.57) (IIIb Clavien) whereas the incidence of foreskin dehiscence was significantly	The postoperative dressing using oxygen-enriched oil-based gel was highly effective, promoting a faster wound healing in patients who underwent distal hypospadias repair. Furthermore, the wound treated with the oxygen-enriched oil-based gel was associated with better postoperative outcome including a lower incidence of foreskin dehiscence and better foreskin retractability compared with the control group. It was also cost-effective and clinically safe since no patients in our series suffered from allergy or intolerance to the product.

							lower in G1 [0/57 (0%)] compared with G2 [5/57 (8.7%)] (*p* = 0.001) (IIIb Clavien).<br><br>Regarding the cost analysis, although the oxygen-enriched oil-based gel medication was more expensive compared with hyaluronic acid cream (23 vs 18 eur), this difference was overcome by the advantages related to the physical characteristics of the viscosity of the ozone oil allowing to adopt a very little amount of the product at each application, whereas the hyaluronic acid cream was aqueous based and not greasy and required a greater amount of the product at each use. We demonstrated the cost-effectiveness of ozone medication; in fact, to accomplish a 3-weeks post- operative treatment, G1 patients needed median two 5-mL syringes of the oxygen-enriched oil-based gel with a cost of 46 eur compared with G2 patients who needed median three 30-mL tubes of hyaluronic acid cream with a cost of 54 eur (*p* = 0.001).	
Positive ROS (Reactive Oxygen Species) Modulator Engineered Device Support Skin Treatment in Locally Advanced Breast Cancer (LABC) Enhancing Patient Quality of Life	Casella D., Palumbo P., Sandroni S., Caponi C., Littori F., Capuano F., Grimaldi L., Marcasciano M., Cuomo R.<br><br>2021	To evaluate the efficacy of a medical device consisting of a polyester scaffold enriched with an oleic matrix with controlled release of ROS in the management of Locally advanced breast cancer (LABC) skin lesions.	Prospective observational study	20 patients/outpatient	Application of oxygen-enriched oleic matrix gel to the wound surface and a preformed cup device in polyester with polyurethane soaked in an oxygen-enriched oleic matrix	A total of twenty (20) patients were included and divided into two different groups (Group A; Group B) of 10 patients each, in sequential order.	After 30 days of treatment, all patients in Group A reported a general improvement in local conditions with a reduction in ulceration area, exudate and odour. The areas of necrosis was calculated to be 60% reduced (178,6-62,3 Group A, 182,3-110,3 Group B). The evaluation of the quality of life index shows a clear improvement in the Group A (18,32-9,7) compared to Group B (19,1-13,7). The evaluation of the pain reported a reduction in the Group A (7,8-2,6) compared to Group B (7,9-5,2).	The results obtained in this preliminary study, although numerically and statistically insignificant, suggests that the application of the abovementioned devices in the setting of LABC skin lesions with ulcerated and neoplastic tissue, represents a safe and effective alternative to traditional dressings and does not lead to the onset of negative effects due to the release of ROS.
Pediatric Endoscopic Pilonidal Sinus Treatment (PEPSiT) in Children With Pilonidal Sinus Disease: Tips and Tricks and New	Esposito C., Mendoza-Sagaon M., Del Conte F., Cerulo M., Coppola V., Esposito G., Cortese G.,	To evaluate the outcome of our new structurated protocol, including PEPSiT, laser epilation, and oxygen-enriched oil-based gel dressing, for treatment of PSD in pediatric patients and describe tips and tricks of the technique.	Retrospective analysis	127 pediatric patients/ postoperative, outpatient	Oxygen-enriched oil-based gel dressing in the first group (G1) and silver sulfadiazine spray in the second group (G2).	We retrospectively reviewed the data of 127 pediatric patients, who underwent PEPSiT for PSD in our institutions over a 36-month period. All patients received laser epilation (LE) before and after surgery. Post-operative dressing was performed using silver sulfadiazine spray and in the last 18 months oxygen-enriched oil-based gel. We	The two groups were compared regarding success rate, recurrence of disease, wound infection rate, wound healing time, post-operative outcome, time to full daily activities and patient satisfaction.<br><br>Furthermore, G1 reported a faster wound healing (average 21 days) compared with G2 (average 29 days) [p = 0.001] and a higher patient satisfaction score (average 4.9) compared with G2 (average 4.2) [p = 0.001].	Our results showed that use of oxygen-enriched oil-based gel in PSD was associated with a significantly faster wound healing and lower recurrence rate compared with our previous protocol based on silver sulfadiazine spray dressing.

Structurated Protocol	Crocetto F., Montaruli E., Escolino M.<br><br>2020					divided the patients in two groups according to the protocol adopted: G1 (laser + oxygen-enriched oil-based gel dressing) included 72 patients and G2 (laser + silver sulfadiazine spray dressing) included 55 patients. The two groups were compared regarding success rate, recurrence, wound infection rate, wound healing time, post-operative outcome, time to full daily activities and patient satisfaction.		
